# PCA3 and TMPRSS2-ERG: Promising Biomarkers in Prostate Cancer Diagnosis

**DOI:** 10.3390/cancers2031432

**Published:** 2010-07-06

**Authors:** Maciej Salagierski, Jack A. Schalken

**Affiliations:** 1 Department of Urology, Radboud University Nijmegen Medical Centre, Nijmegen, The Netherlands; 2 First Urology Department, Medical University of Łódź, Poland; E-Mail: maciej.salagierski@umed.lodz.pl

**Keywords:** prostate cancer, diagnosis, molecular, marker, *PCA3*, *TMPRSS2:ERG*

## Abstract

The search for the biomarkers to precisely and non-invasively characterize the biology of prostate cancer (PCa) is the focus of many laboratories across the world. Although prostate-specific antigen (PSA) remains the standard diagnostic tool for PCa, its low specificity leads to unnecessary biopsies in a substantial number of patients. More importantly, with the current status of knowledge, it is very difficult to early identify individuals with a life-threatening disease who require an immediate treatment. The significant advances in genetics and biotechnology in recent years has led to the discovery of new molecular markers including *PCA3* and the *TMPRSS2:ERG* genomic fusion. Both *PCA3* and *TMPRSS2:ERG*, compared to PSA, show an increased specificity in PCa detection. However, the quest for a single PCa marker that can fully satisfy urologists and their patients is still ongoing. The aim of this review is to present the recent findings on *PCA3* and *TMPRSS2:ERG* and to describe their clinical implications and performance.

## 1. Introduction

Prostate cancer (PCa) constitutes the most common cancer in men with a steadily rising incidence affecting on average one in six men during their lifetime [1]. The increase in morbidity is related to the increasing overall life expectancy, more frequent prostate-specific antigen (PSA) testing and larger number of men undergoing biopsy. Unfortunately, currently used diagnostic tools are rarely able to determine the progression of PCa or to predict its clinical outcome. As a consequence, it is not only difficult to identify a life-threatening early stage disease requiring rapid radical treatment, but also large numbers of patients with indolent tumors are over-treated, *i.e.*, receiving unnecessary inadequate and costly therapies that do not result in a survival benefit. The significance of prostate-specific antigen (PSA) by itself as a standard diagnostic tool in PCa has been recently frequently questioned due to its diagnostic limitations, mainly its low positive predictive value (PPV). A PPV of PSA within its ‘gray zone’ between 4.0 and 10.0 ng/mL, ranges from 25 to 40%, resulting in an elevated, up to 75%, negative biopsy rate [2]. Repetition of the first negative biopsy, although strongly recommended in cases of PSA elevation, does also not perform well. Therefore, there is an urgent need for the development and implementation of new markers with improved specificity in PCa detection, both being able to identify patients with an early aggressive or clinically significant disease and to determine its prognosis.

Markers research has been the focus of many laboratories across the world. Within the last decade, due to a significant advance in genetics and cell biology, new emerging molecular markers have been widely investigated [3]. Their appearance has been the natural consequence of recently described genetic changes in PCa including gene fusions and messenger RNA (mRNA) alterations. Several worth reporting biomarkers have been identified and a great amount of work has been done so far in an effort to develop new assays based on the recent findings. The only marker that has been introduced into clinic remains Prostate Cancer Gene 3 (*PCA3*). A very promising and highly prostate cancer specific marker is the fusion transcript *TMPRSS2:ERG*. However, the heterogeneity of the disease among the affected individuals, multifocality and marked variability in the progression of PCa makes implementation of new biomarkers complex. Our objective is to present recent findings on the application of *PCA3* and *TMPRSS2:ERG* in prostate cancer diagnosis and management. The clinical implications and performance of biomarkers will be also reviewed. 

## 2. PCA3

### 2.1. Structure and Specificity

*PCA3* is a non-coding RNA and the most specific clinically available prostate malignancy marker described so far: *PCA3* RNA expression is restricted to prostate, *i.e.*, it is not expressed in any other normal human tissue nor in any other tumor [6,7]. The presence of *PCA3* RNA was firstly reported by Bussemakers *et al.* in 1999. The gene encoding *PCA3* is located on chromosome 9q21–22 and consists of four exons [4]. The most frequent transcript contains exons 1, 3, 4a and 4b. Since its discovery, the structure of *PCA3* has been widely investigated, and most recently four new transcription start sites as well as two additional exon 2 sequences and four additional polyadenylation sites have been described [5]. The *PCA3* RNA is highly overexpressed in 95% of tumors compared to normal or benign hyperplastic prostate tissue. Hessels *et al*. reported a median 66-fold upregulation of *PCA3* in PCa tissue compared with normal prostate tissue. In addition, an average 11-fold upregulation was revealed in prostate tissue specimens containing less than 10% of PCa cells [6]. Several years following *PCA3* identification, an assay that detects *PCA3* in urine has been developed. 

### 2.2. *PCA3* Assay and *PCA3* Score

Apart from being highly sensitive and specific, a biomarker should be easily detectable with non-invasive methods, e.g., using blood or urine samples. As cancerous cells with high levels of *PCA3* are shed from the prostate into the urine, the levels of *PCA3* RNA can be measured not only in prostate tissue specimens but also in urine and the urinary sediments after prostatic massage. The collection of 20–30 mL of voided urine after a digital rectal examination (DRE) (three strikes per prostate lobe) is required to perform the test. The only assay available commercially- APTIMA PCA3 test (Gen-Probe Incorporated, San Diego, CA, USA) quantitatively detects the expression of *PCA3* RNA in urine and prostatic fluids using transcription-mediated amplification [7]. In order to assess the probability of PCa detection on prostate biopsy, the quantitative PCA3 score was developed. The PCA3 score is defined as *PCA3*-RNA/PSA-mRNA ratio, meaning that *PCA3* expression is normalized with the PSA expression used as a housekeeping gene. 

### 2.3. *PCA3* Score for Biopsy Making Decisions

The PCA3 score correlates with the likelihood of positive biopsy and by that means helps clinicians in biopsy making decisions. The higher the PCA3 score the greater the probability of a positive biopsy. Deras *et al.* reported a 14% positive biopsy rate for men with a PCA3 score <5 *versus* almost 70% positive biopsy rate for PCA3 scores over 100 [8]. As the PCA3 score of 35 yielded the greatest diagnostic utility, demonstrating the optimal balance between specificity and sensitivity, it has been adopted as a cut-off score. The average sensitivity and specificity of the PCA3 urine test is relatively high—66% and 76%, respectively (*vs.* 47 % specificity for serum PSA level) [9]. Haese *et al.*, on a group of 463 patients, showed that men with a PCA3 score over 35 have a 39% chance of a positive repeat biopsy compared to 22% likelihood in men with a PCA3 score lower than 35 [10]. This study confirmed that the PCA3 score is significantly higher in men with a positive biopsy (median value of 33.7) than in men with a negative biopsy (median value of 19.5). Moreover, the negative predictive value of the test is very high, reaching—depending on a PCA3 cut-off value—90%. The authors reported that when using a threshold PCA3 score of 20 in their group, a 44% reduction of repeat biopsy could be achieved while missing only 9% of clinically significant cancers (Gleason score ≥ 7). 

### 2.4. *PCA3*—A Correlation with Gleason Score and Pathological Findings

As the results of different studies are conflicting, the questionable aspect of PCA3 score remains its ability to assess PCa aggressiveness or its clinical behavior. The discrepancies between studies are most probably related to cohort differences, different end points, *etc*.

In one of the recently published papers, Hessels *et al.* did not reveal significant association between PCA3 score in urinary sediments after DRE (see PCA3 assay) and any of PCa prognostic parameters including Gleason grade, tumor volume and tumor stage [11]. Those results are in agreement with the previous study of van Gils *et al.* [12]. Likewise, no correlation was found between *PCA3* expression in PCa tissue and Gleason score [13]. Conversely, Nakanishi *et al.* have described a relationship between the preoperative PCA3 score and radical prostatectomy specimen’s tumor volume. The average PCA3 score was statistically lower in low volume (less than 0.5 cc) tumors. This study also showed that increasing PCA3 score was associated with a higher Gleason score [14]. Marks *et al.* supported the findings of Nakanishi *et al.* demonstrating the presence of a higher PCA3 score in men with a Gleason score ≥ 7 [15]. Importantly, Whitman *et al.* observed that *PCA3* detected in the post-DRE urine of patients with PCa correlated with pathological findings, *i.e.*, extracapsular extension and tumor volume [16]. 

### 2.5. Clinical Implications of *PCA3* Score

Advantageously for diagnostic purposes, unlike PSA concentration, PCA3 score is independent of prostate volume, the number of prior biopsies and unaffected by patients’ age. Most importantly, the test is not influenced by principal causes of non-cancerous PSA elevations, *i.e.*, benign prostatic hyperplasia (BPH) and prostatitis. Thus, the PCA3 assay, being more specific than serum PSA, appears extremely useful for detecting the presence of PCa in men with frequently observed alternative causes of PSA elevation, including inflammation of the gland and increase of its size. Conversely, in patients with normal or low (<4 ng/mL) PSA values, *PCA3* could constitute a valuable tool in PCa diagnosis, enabling detection of clinically relevant tumors. It appears that *PCA3* expression is unaffected by the pharmacotherapy of the gland including the application of type I and II 5-α reductase inhibitors. Further, *PCA3* seems very helpful for establishing treatment decisions in PCa patients undergoing active surveillance by helping clinicians to select individuals requiring rapid treatment, e.g., demanding prompt surgery or radiotherapy.

In addition, PCA3 test allows detection of the precancerous lesion known in PCa as high grade prostatic intraepithelial neoplasia (HGPIN). Popa *et al.* demonstrated that over 90% of HGPIN tissue expressed *PCA3* [17]. Another study by Haese *et al.* demonstrated that PCA3 score in HGPIN patients was 16% higher than in tissue of men without this lesion [10]. As a consequence, the PCA3 test might be helpful in monitoring patients with the presence of HGPIN, *i.e.*, an increase in PCA3 score would trigger biopsy decision. Thus, clinical application of the PCA3 assay appears much broader than only testing men with an elevated PSA and a negative biopsy. 

## 3. TMPRSS2: ERG

### 3.1. Mechanism of the Fusion

Genetic alterations are considered as initial events in oncogenesis occurring in almost all human malignancies [18]. Although gene rearrangements are frequent in hematologic malignancies, they have rarely been reported in solid tumors, which account for more than 80% of cancer-related deaths [1]. The discovery of imatinib (Gleevec)—the drug targeting the kinase domain of the *BCR-ABL* fusion (*BCR* gene from chromosome 9 and *ABL* gene of chromosome 22) has revolutionized the management of chronic myeloid leukemia by significantly improving patient’s survival. Therefore, the identification of new cancer specific fusion products might constitute a cornerstone for the implementation of targeted drug therapy not only limited to blood malignancies. In the near future, targeting gene rearrangements might become an important innovative approach in solid tumors management. 

In recent studies gene rearrangements involving androgen regulated gene *TMPRSS2* (trans-membrane protease, serine 2) and *ETS* transcription factor genes (*ERG*, *ETV1*, *ETV4 and ETV5*) have been identified in PCa patients. Importantly, *ERG* is currently considered as a key oncogene in PCa [19]. *TMPRSS2:ERG* fusion constitutes the most common variant, occurring in approximately 50% of PCa patients. Both fusion partners map to chromosome 21; *TMPRSS2* at 21q22.3 and *ERG* at 21q22.2. The predominant mechanism for gene fusion is the loss of 2.8 Mb of genomic DNA between *TMPRSS2* and *ERG* [20]. Considering the high prevalence of PCa, *TMPRSS2:ERG* fusion is the most common genetic aberration described so far in human malignancies [21]. Interestingly, the gene rearrangement occurs almost exclusively (in 95% of cases) in patients with marked overexpression of *ERG*. 

### 3.2. Clinical Implications of *TMPRSS2: ERG*

*TMPRSS2:ERG* rearrangement can be, similarly to the *PCA3* gene, detected in urine after DRE [22]. The detection of *TMPRSS2:ERG* fusion in urine as described by Hessels *et al.* has an over 90% specificity and 94% positive predictive value for PCa detection [22]. Given the high specificity of the test, fusion status in *ERG* positive men may soon serve in the clinic as a viable biomarker for establishing the presence or absence of PCa [21]. The *TMPRSS2:ERG* fusion product can be found in 20% of prostatic intraepithelial neoplasia (PIN) adjacent to fusion positive cancer tissue but not in benign prostate tissue specimens or proliferative inflammatory atrophy (PIA) [23]. 

The data on the association between *TMPRSS2:ERG* fusions and patients outcome remain conflicting. Several studies suggest that *TMPRSS2:ERG* fusion prostate cancer contribute to a more aggressive cancer phenotype, which is associated with higher tumor stage and prostate cancer-specific death [24,25]. Demichelis *et al.*, in the group of 252 patients under active surveillance for T1a-b, Nx, M0 tumors with a median follow-up of 9.1 years, demonstrated that the fusion transcript was associated with metastases and lethal prostate cancer [19]. The fusion-positive tumors had also significantly higher Gleason scores. However, in the mentioned study, the *TMPRSS2:ERG* fusion product was identified only in 15% of PCa patients, which is much less than found by other authors. Conversely, the recently published study by Gopalan and his colleagues [26] showed no correlation between *TMPRSS2:ERG* rearrangement and outcome in patients treated with radical prostatectomy. In this study, the clinical association between the presence of fusion transcript and outcome was analyzed on a large group of 521 PCa patients. The presence of the fusion product was even associated with lower PCa stage. Interestingly enough, the aggressive tumor behavior, *i.e.*, metastatic disease, was associated with a copy number increase of *TMPRSS2:ERG* loci on chromosome 21. In the most recently published study, Rubio and colleagues reported no association between fusion gene status and prostate cancer clinicopathological characteristics. Although the presence of the *TMPRSS2:ERG* translocation was not related to biochemical or clinical progression, the authors were able to identify two groups of patients with different prognosis of PCa based on the presence or absence of the fusion [27]. Importantly, Clark *et al.* demonstrated that the best diagnostic performance in predicting prostate biopsy outcome might be achieved by combining *TMPRSS2:ERG* with PSA and DRE [28].

### 3.3. Combination of *PCA3* with *TMPRSS2: ERG*

Currently, we are still unable to discriminate between clinically important and indolent prostate malignancies, e.g., a low PCA3 score does not exclude a clinically significant tumor. Therefore, some authors are trying to evaluate whether the specificity and sensitivity of PCa detection could increase by combining novel molecular biomarkers. Hessels *et al.* analyzed the urinary sediments of 108 men for the presence of both *PCA3* and *TMPRSS2:ERG* products, and showed that by combining two assays the sensitivity of PCa detection markedly increases (from 63% for *PCA3* alone to 73% for both tests) without compromising the specificity [22]. By that means, the very satisfying diagnostic performance of PCa detection was achieved. Similar findings were reported by Laxman *et al.*, who by incorporating four urine biomarkers, including *PCA3* and *TMPRSS2:ERG*, achieved a 65.9% specificity and 76.0% sensitivity in PCa detection. This quantitative multiplex biomarker study also outperformed *PCA3* alone in the detection of PCa [29]. Petrovics *et al.* demonstrated a very promising gene panel for PCa diagnosis by investigating a marker panel composed of *ERG*, *PCA3* and the alpha-methylacyl-CoA racemase (*AMACR*). The study showed that at least one of the three genes included in the panel was overexpressed in almost all examined PCa specimens (54 of 55) [30]. This kind of innovative approach for the development of a urine multiplex test may be an important and necessary step (considering the heterogeneity of PCa) in creating a marker panel with a very high accuracy in PCa detection and in predicting prognosis of the disease [31].

## 4. Conclusions

Taken together, PSA testing revolutionized the management of PCa in the last century. However, considering the well-known limitation of PSA in cancer detection, especially its low specificity, the development of new markers are necessary. With the current status of knowledge, PCA3 assay might be a useful tool for biopsy making decisions to helps urologists avoid unnecessary biopsies, as well as serving as a very good test for screening patients with unspecific causes of PSA elevations. Despite very promising data, further investigations and prospective clinical trials are required mainly to assess prognostic performance of PCA3 score. The presence of positive prostate biopsy findings despite the negative *PCA3* result (low PCA3 score does not exclude PCa) [10] doesn’t support PCA3 assay as sufficient for PCa management. The combination of *PCA3* and *TMPRSS2:ERG* assays by significantly improving diagnostic performance might become extremely helpful in therapeutic decisions. Further, new emerging molecular markers might soon lead to both a decrease in patients morbidity related to current PCa therapies and to the reduction of the cost of PCa management.
